# Intraoral Microbial Metabolism and Association with Host Taste Perception

**DOI:** 10.1177/0022034520917142

**Published:** 2020-05-20

**Authors:** A. Gardner, P.W. So, G.H. Carpenter

**Affiliations:** 1 Salivary Research, Centre for Host-Microbiome Interactions, Faculty of Dental, Oral & Craniofacial Sciences, King’s College London, London, UK; 2 Department of Restorative Dentistry, Dental Hospital and School, University of Dundee, Dundee, UK; 3 Department of Neuroimaging, Institute of Psychiatry, Psychology and Neuroscience, King’s College London, Maurice Wohl Clinical Neuroscience Institute, London, UK

**Keywords:** saliva, metabolomics, microbiota, microbiology, biofilm, proteins

## Abstract

Metabolomics has been identified as a means of functionally assessing the net biological activity of a particular microbial community. Considering the oral microbiome, such an approach remains largely underused. While the current knowledge of the oral microbiome is constantly expanding, there are several deficits in knowledge particularly relating to their interactions with their host. This work uses nuclear magnetic resonance spectroscopy to investigate metabolic differences between oral microbial metabolism of endogenous (i.e., salivary protein) and exogenous (i.e., dietary carbohydrates) substrates. It also investigated whether microbial generation of different metabolites may be associated with host taste perception. This work found that in the absence of exogenous substrate, oral bacteria readily catabolize salivary protein and generate metabolic profiles similar to those seen in vivo. Important metabolites such as acetate, butyrate, and propionate are generated at relatively high concentrations. Higher concentrations of metabolites were generated by tongue biofilm compared to planktonic salivary bacteria. Thus, as has been postulated, metabolite production in proximity to taste receptors could reach relatively high concentrations. In the presence of 0.25 M exogenous sucrose, increased catabolism was observed with increased concentrations of a range of metabolites relating to glycolysis (lactate, pyruvate, succinate). Additional pyruvate-derived molecules such as acetoin and alanine were also increased. Furthermore, there was evidence that individual taste sensitivity to sucrose was related to differences in the metabolic fate of sucrose in the mouth. High-sensitivity perceivers appeared more inclined toward continual citric acid cycle activity postsucrose, whereas low-sensitivity perceivers had a more efficient conversion of pyruvate to lactate. This work collectively indicates that the oral microbiome exists in a complex balance with the host, with fluctuating metabolic activity depending on nutrient availability. There is preliminary evidence of an association between host behavior (sweet taste perception) and oral catabolism of sugar.

## Introduction

Microbial sequencing technologies greatly advanced knowledge of the diversity of the human oral microbiome. Complementary technologies such as metabolomic profiling likely represent an equally valuable, yet underused, source of information (Takahashi 2015). Metabolomic profiling of oral fluid, particularly saliva, generally focuses on biomarker discovery. Metabolomic profiling of saliva appears to offer diagnostic promise for oral cancer (Sugimoto et al. 2010; Wang et al. 2014; Ishikawa et al. 2016), periodontal disease (Aimetti et al. 2012), dental caries (Fidalgo et al. 2013; Pereira et al. 2019), and primary Sjögren syndrome (Mikkonen et al. 2013; Kageyama et al. 2015). Saliva has also been implicated as a source of biomarkers for systemic diseases, including breast and prostate cancer (Sugimoto et al. 2010) and neurological disease (Figueira et al. 2016; Yilmaz et al. 2017). These studies typically focus on finding differences between disease and control samples. Investigation of why these metabolic differences arise is lacking. An important step in enhancing the knowledge of the oral microbiome is moving from simply determining what microorganisms are present to determining the significance of their net metabolomic activity (Takahashi 2015). Study of the human gut microbiome and metabolome is unveiling important insights into the symbiotic relationship between the microbiome and their host, in health and disease (Hirata and Kunisawa 2017; Liu et al. 2017; Santoru et al. 2017; Zierer et al. 2018).

There remain important gaps in the collective knowledge of the salivary metabolome. Unlike for the gut, the link between the salivary metabolome and microbiome remains sparsely studied. Prominent gut metabolites, including acetate, propionate, and butyrate, collectively termed short-chain fatty acids (SCFAs), arising from the bacterial fermentation of dietary fiber are known to confer health benefits. These include maintenance of epithelial barrier integrity, immune signaling, and anticancer regulation (Morrison and Preston 2016; Rooks and Garrett 2016). SCFAs are also prominent salivary metabolites. Until recently, a significant question concerned the origin of salivary metabolites and the extent to which they are derived from the host or the microflora. It has recently been demonstrated that parotid saliva (PS) is free of SCFAs, aside from trace levels of acetate. Furthermore, the SCFA concentration of whole-mouth saliva (WMS) correlates strongly with the oral microbial load (Gardner et al. 2019).

Glandular saliva as measured by nuclear magnetic resonance spectroscopy (^1^H-NMR) is relatively sparse in metabolite content compared to WMS. Many other metabolites present in WMS appear to be largely of microbial origin, including amines (methyl, dimethyl, and trimethylamine) and amino acids such as phenylalanine and glycine. The few high-concentration metabolites present in glandular saliva include citrate, lactate, and urea. Salivary urea is consumed by oral bacteria postsecretion, and WMS urea concentrations correlate inversely with both microbial load and plaque abundance (Gardner et al. 2019; Liebsch et al. 2019). Lactate is arguably the most familiar salivary metabolite among dental professionals. The role of lactate production from fermentable carbohydrates in demineralizing tooth tissue has been known for 80 y (Miller et al. 1940). While other organic acids have also been implicated in the caries process (Silwood et al. 1999), lactate reaches the highest concentrations following oral exposure to fermentable sugars. Nevertheless, under resting conditions without recent (>1-h) exposure to oral carbohydrates, WMS lactate concentrations are not elevated above PS concentrations and are less concentrated than circulating blood lactate concentrations (Gardner et al. 2019). These shifts in metabolism upon exposure to exogenous nutrients underpin a major difference between the gut and oral metabolomes. Whereas gut microbes have a continual source of nutrients in the form of dietary fiber, oral microbial communities must endure significant periods of time without exposure to nutrients consumed by the host. Sleep would likely represent the longest time period for oral bacteria to subsist without oral exposure to exogenous nutrients. However, the saliva initially produced on waking has been demonstrated to be richer in many metabolites, including amines and SCFAs, relative to saliva collected throughout the day (Wallner-Liebmann et al. 2016). Therefore, oral bacteria must be capable of metabolizing additional substrates.

Another emerging theme from the growing literature on the gut microbiome is that the microbiome manipulates host behaviors via their metabolic activity. A complex relationship is being unveiled, linking dietary choices with the microbial metabolism of consumed foods by gut bacteria via molecular signaling that influences host satiety response. Such a relationship essentially forms a feedback cycle where the consumption of unhealthy, processed foods ultimately leads to a desire to consume more of the same foods, resulting in adverse metabolic consequences such as obesity and associated conditions (Sandhu et al. 2017). These host-microbiome interactions have led to gut bacteria being described as “microscopic puppetmasters” (Alcock et al. 2014). Action on taste receptors has been identified as a possible mechanism of such microbial manipulation of their host, but the literature directly supporting this is currently limited to animal models (Alcock et al. 2014). More important, despite being focused on oral sensory processes, the emphasis of these animal studies is the gut microbiome. It would seem logical that the oral microbiome would be a more appropriate target when investigating microbial impairment of taste function. It has been hypothesized that metabolic activity of tongue biofilms local to taste receptors may be critical in generating metabolites that modulate individual sensory perception (Neyraud and Morzel 2019). The pattern of bacterial substrate utilization and metabolite output relevant to these processes is unclear, although catabolism of exogenous nutrients is implicated.

The aim of this work is therefore to explore whether the net metabolic activity of oral microflora might influence host taste perception, particularly in the presence and absence of exogenous nutrients. The pattern of metabolites arising from saliva catabolism by tongue biofilm and WMS bacteria was first established in vitro, modeling a fasted state such as sleep. Subsequently, in vivo catabolism of exogenous sucrose was analyzed with respect to host taste sensitivity to a sucrose challenge, modeling carbohydrate intake.

## Materials and Methods

### Ethical Approval

Work was conducted following approval from King’s College London ethics committee (HR-15/16–2508). All volunteers provided written consent.

### Investigation of Oral Microbial Metabolism in the Absence of Exogenous Nutrients In Vitro

#### Sample Collection

PS (20 mL) was collected from a single volunteer using a sterilized Lashley cup and 1% mass/volume food-grade tartaric acid stimulation (Gardner et al. 2019). PS was filtered through a 0.2-µm filter, aliquoted (500 µL) into sterile tubes, and stored at −80°C for 1 wk prior to use.

Bacterial inoculums were sourced from 6 healthy adult volunteers 1 h after eating, drinking, or oral exposure to exogenous substances. Dietary information was not gathered. Antibiotic use in the preceding 6 mo and active oral disease (based on visual examination by a dentist) were exclusion criteria. Unstimulated WMS was collected from each volunteer. Biofilm samples from the anterior and posterior tongue were collected using sterilized, preweighed plastic scrapers (cat. 231-0639; VWR). The location of sample was based on proximity to circumvallate papillae posteriorly and fungiform papillae anteriorly.

#### Inoculation and Incubation Conditions

PS aliquots were thawed on ice. Aliquots were inoculated with 20 µL WMS or 20 mg of tongue biofilm from either tongue site (i.e., 4% by volume/mass, respectively). Control samples were prepared with 20 µL sterile phosphate-buffered saline (PBS). Inoculated sample tubes were stored inside Sterilin universal tubes (Thermo Fisher Scientific) with wet tissue paper in the bottom to minimize evaporative fluid loss. Samples were incubated at 37°C for 24 h in an anaerobic cabinet with gas blend of 10% H_2_, 10% CO_2_, and 80% N_2_. Tube lids were pierced with sterilized forceps to allow gaseous exchange.

Nonincubated control samples were prepared immediately prior to analysis. One control was PS mixed with 4% PBS to control for any effects of incubation alone in the absence of bacteria. The second control was PS mixed with 20 µL pooled WMS (4%) to control for any compositional changes arising from the baseline metabolites present in the WMS inoculum. The experimental design is summarized in Appendix Figure 1.

#### Sample Analyses

Postincubation bacterial load, protein composition, and metabolite composition were analyzed as described previously (Gardner et al. 2018, 2019; Gardner and Carpenter 2019), respectively. A brief overview is presented in the appendix material.

### Investigation of Oral Microbial Metabolism in the Presence of Exogenous Nutrients and Associations with Host Taste Sensitivity In Vivo

#### Sample Collection and Study Design

Food-grade sucrose solutions (Sigma) were prepared at 0.25 M in Buxton (Nestle) mineral water. Experiments were conducted between 2:00 and 3:00 p.m., at least 1 h after the last exposure to exogenous substances. Volunteers were administered 10 mL of mineral water as a control and instructed to passively hold the liquid in the floor of the mouth for 30 s. The water was expectorated and WMS collected into preweighed universal tubes over 2 min. This process was repeated with 0.25 M sucrose.

Participants rated their maximum perceived intensity of the sucrose sweetness on generalized labeled visual analog scales (glVASs; Appendix Fig. 3). Participants were first familiarized with the use of the scale via verbal and written instructions. Fifty-two participants were screened for taste sensitivity. Inclusion/exclusion criteria were as described for biofilm donors and included no reported deficiency in salivary flow or taste function. Salivary samples from sensitive and relatively insensitive sucrose perceivers, defined as rating sweetness as greater or less than 1 standard-deviation from the mean, were selected for further analysis, (*n* = 9 per group). Conformity with Strengthening the Reporting of Observational Studies in Epidemiology (STROBE) guidelines (ISPM, Bern, Switzerland) for case-control studies was ensured.

#### Salivary Analyses

Salivary flow rate was calculated in g/min by dividing the mass of saliva collected by the collection time. Samples were analyzed by ^1^H-NMR spectroscopy as described and targeted metabolite concentrations were quantified. Biofilm metabolite output was calculated in µmol/min by multiplying metabolite concentration by flow rate. Differences in metabolite output relative to control following sucrose exposure were compared between the sensitive and relatively insensitive perceivers. Relative flow rate changes and relevant metabolite ratios were also determined and compared. Lactate/pyruvate ratios were calculated by dividing lactate output by pyruvate output and citrate/pyruvate ratios calculated by dividing citrate output by pyruvate output.

### Statistical Analyses

Data were primarily analyzed in GraphPad Prism 8 (GraphPad Software) and Knime v.3.4.2 (KNIME). Following inspection for normality (Shapiro-Wilk test and Q-Q plots), data were analyzed by appropriate statistical tests, including analysis of variance (ANOVA), single-sample *t* test, and 2-tailed paired *t* tests. Colony-forming unit (CFU) densities were logarithmically transformed prior to analysis. Adequate statistical power was confirmed post hoc for the differences observed in the in vivo study.

## Results

### Oral Bacteria Consume Salivary Proteins

**Figure 1.**
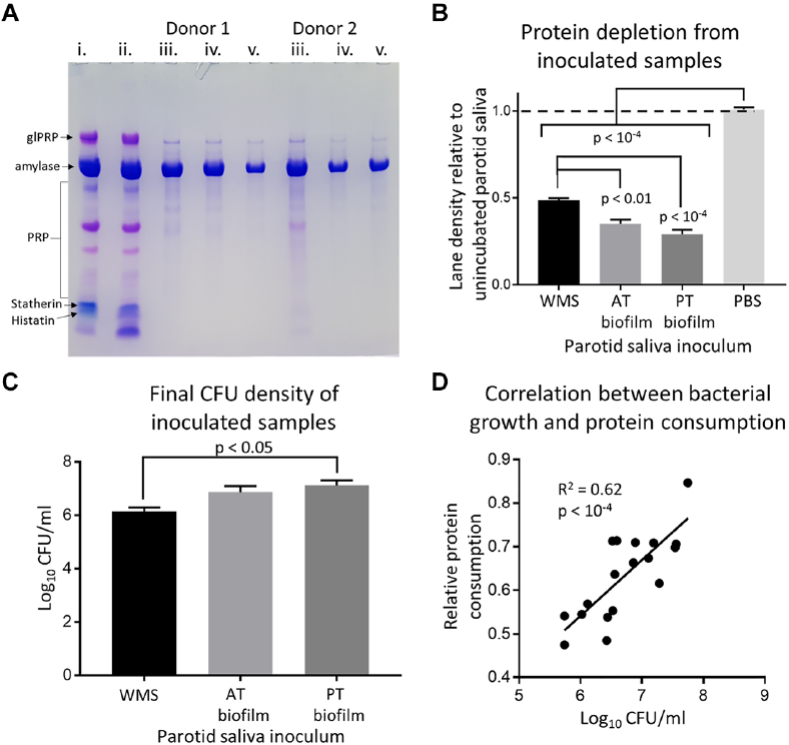
Summary of bacterial protein consumption from parotid saliva (PS). (**A**) Coomassie-stained polyacrylamide gel. Lane i = unincubated PS; ii = phosphate-buffered saline (PBS) inoculated, incubated parotid saliva; iii = whole-mouth saliva (WMS) inoculated PS; iv = anterior tongue biofilm inoculated PS; v = posterior tongue biofilm inoculated PS. Samples from 2 representative donors are shown on this gel. glPRP, glycated proline-rich protein; PRP, proline-rich proteins. (**B**) Protein consumption from the inoculated samples, measured relative to the unincubated PS (dotted line). AT, anterior tongue; PT, posterior tongue. (**C**) Final log_10_ colony-forming units (CFU)/mL from the inoculated samples. Data in B and C are mean ± SEM, analyzed by Tukey’s multiple comparison test following analysis of variance (*n* = 6 samples per group). (**D**) Correlation between protein consumption and final log_10_ CFU/mL from all inoculated samples, *n* = 18, measured by Pearson’s correlation.

PS was minimally affected by incubation alone, displaying minor changes in statherin and low molecular weight (MW) proteins. Inoculated samples universally showed considerable protein loss, with amylase typically the only residual protein. Lane density of samples was significantly reduced relative to controls for all inoculums. Tongue biofilm samples had significantly reduced protein relative to WMS inoculated samples. Log_10_ CFU of inoculated samples differed significantly between WMS and posterior tongue biofilms. A moderate correlation (*R* ^2^ = 0.62) was found between final CFU and protein consumption of the samples. These results are summarized in Figure 1.

### Oral Bacteria Generate Metabolites from Parotid Saliva

**Figure 2.**
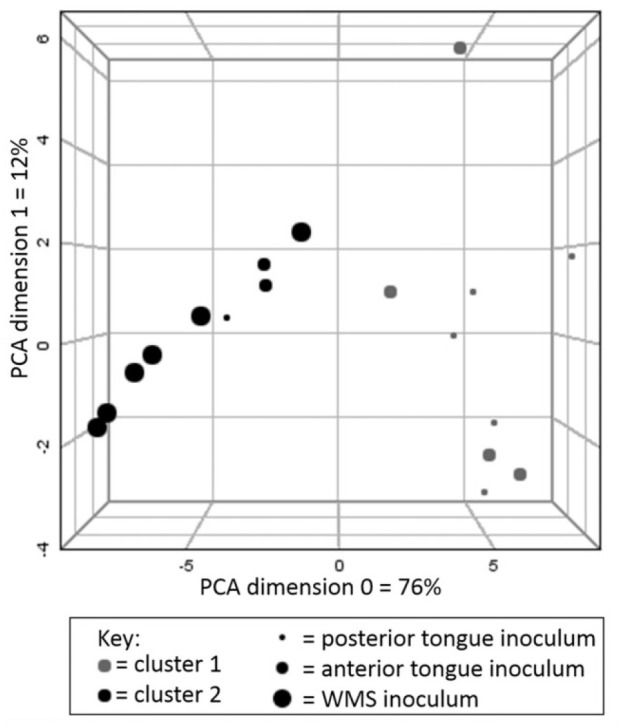
Principal component analysis (PCA) plot with *k*-means cluster analysis of metabolite profiles of inoculated parotid saliva (PS). Statistical clusters are indicated by color, and inoculum is indicated by size. There appears to be a degree of separation between whole-mouth saliva (WMS) and tongue biofilm inoculums, with only 1 posterior tongue and 2 anterior tongue samples being clustered with WMS inoculums. There is no evidence of a distinction between posterior and anterior tongue biofilm inoculums.

Metabolic content of PS inoculated with oral bacteria was considerably different from PBS inoculated PS. Typical spectra pre- and postincubation are shown in Appendix Figure 4. Changes in the metabolite concentrations are shown in Appendix Table 1. The majority of host-derived metabolites present in PS at baseline were partly or wholly consumed by oral bacteria. Inoculated samples displayed considerable concentrations of SCFAs, amino acids, and phenolic compounds. These tended to be most concentrated in the tongue biofilm inoculated samples. Spectral profiles of control samples were not significantly different from the baseline unincubated PS. The only measured metabolic difference between control and baseline parotid saliva was that phenylalanine was not detected in baseline PS. Multivariate analysis found that there was a degree of separation between the metabolic composition of tongue biofilm and WMS inoculated samples (Fig. 2). The consumption of proteins correlated with the generation of several metabolites, notably acetate, butyrate, propionate, and phenylacetate (Appendix Fig. 5). A comparison of interindividual variability in metabolite profiles of inoculated samples and participant WMS found no differences in variation between the samples (Appendix Fig. 6). Importantly, endogenous salivary metabolites were also consumed from the baseline PS. Endogenous glucose was fully consumed in all cases. Citrate and urea were significantly consumed by all inoculums, but pyruvate and lactate were significantly consumed by tongue biofilm but were not significantly consumed by WMS bacteria.

### Host Sensitivity to Sucrose Is Associated with Different Intraoral Bacterial Sucrose Catabolism In Vivo

**Figure 3.**
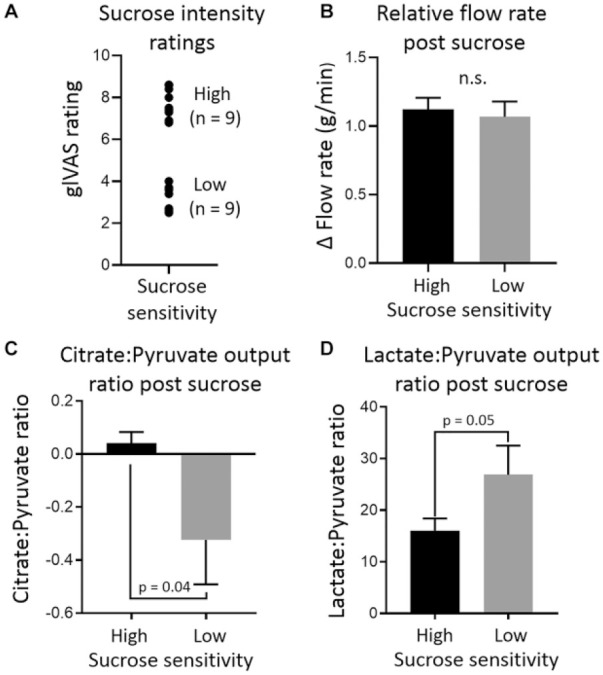
Summary of data comparing microbial sucrose catabolism between high-sensitivity and low-sensitivity sucrose perceivers. (**A**) High- and low-perceiver groupings based on their generalized labeled visual analog scale (glVAS) intensity ratings of 0.25 M sucrose. (**B**) Postsucrose flow rate relative to control for both perceiver groups, which did not differ. (**C**, **D**) The difference between high- and low-sensitivity perceivers for citrate/pyruvate and lactate/pyruvate ratios, respectively. Bar graphs display mean ± SEM; *P* values are for 2-tailed *t* test. Based on the present sample (*n* = 9), a β value of 0.81 was calculated at α = 0.05. n.s., not significant.

Exposure of oral bacteria to sucrose causes significant changes in the salivary concentration and outputs of multiple metabolites (Appendix Table 2). Data subdivided by sucrose sensitivity are presented in Appendix Table 3. When comparing subjects with high and low sensitivity to sucrose, no significant difference in relative salivary flow rate was detected. Significant differences in lactate/pyruvate ratios and citrate/pyruvate ratios between high- and low-sensitivity perceivers were detected. Low-sensitivity perceivers had a significantly lower citrate/pyruvate ratio (–0.32 ± 0.17 vs. 0.04 ± 0.04, respectively) and a significantly higher lactate/pyruvate ratio (26.88 ± 5.63 vs. 16.04 ± 2.35, respectively) compared to high-sensitivity perceivers. Data are presented in Figure 3.

## Discussion

Despite being recognized as an important nutrient source for oral bacteria (Ruhl 2012; Takahashi 2015), microbial metabolism of saliva has been sparsely studied. Carbohydrate moieties of salivary glycoprotein (MUC5B) have been identified as a target for microbial catabolism, but the present results demonstrate that salivary proteins are readily catabolized by oral bacteria in a nonspecific fashion. Amylase was typically the only protein partly remaining at the experimental endpoint. Many of the metabolites generated from the microbial breakdown of salivary protein are present in WMS, including SCFAs, glycine, and phenylalanine. A depiction of relevant metabolic pathways is shown in Appendix Figure 7. A number of additional metabolites were generated in this in vitro model at concentrations above those typically seen in healthy WMS. These included amino acid degradation by-products such as putrescine and 5-aminopentanoate and phenolic compounds such as 3-phenylpropionate and phenylacetate. Phenylacetate has been implicated as a biomarker in periodontal disease (Liebsch et al. 2019). This indicates the importance of ecological niche to the pathogenicity of oral bacteria. While all participants harbored oral bacteria capable of proteolysis and generation of phenylacetate in vitro, these metabolites were not detected in their baseline WMS samples, indicating an environment-dependent shift toward proteolysis.

As predicted by Neyraud and Morzel (2019), the tongue biofilm generated an abundance of metabolites, some of which have the potential to manipulate taste and oral perception. These include SCFAs, which have previously been inversely associated with oral sensitivity to oleic acid (Mounayar et al. 2014). A number of other amino acids with the potential to alter taste perception were also observed. The concentrations of glycine, valine, leucine, phenylalanine, and proline produced following 24-h in vitro incubation, while higher than those generally found in saliva, were still below the respective taste detection thresholds. Nevertheless, local concentrations of such metabolites within tongue biofilm in vivo might theoretically reach higher concentrations (Feron 2019). Interestingly, there were differences between the metabolic patterns of WMS and tongue biofilm inoculums. These differences may be attributable to different bacterial loads (WMS inoculums yielded significantly lower log_10_CFU/mL than posterior tongue biofilms). This might explain quantitative differences, but qualitative differences in spectral profile of WMS and tongue biofilm inoculated samples were detected by principal component analysis (PCA). Therefore, microbial compositional differences between the inoculum sources, as well as the planktonic nature of WMS and biofilm structure of tongue samples, may be more important in shaping the net metabolic activity of oral bacterial niches. Differences in endogenous metabolite consumption between WMS and tongue biofilm inoculums were also found, in particular relating to lactate and pyruvate consumption. Alongside the in vivo findings, this highlights the complexity of host-microbiome interactions in the oral cavity. For example, salivary lactate concentrations are in constant balance between delivery rate from host-glandular fluid, microbial consumption under fasted conditions, and microbial generation upon exposure to exogenous nutrient sources. There are several limitations of this in vitro study. First, the experimental design represents a static nutrient pool, whereas even during sleep, when salivation is minimal, a degree of flux would occur in the oral cavity. Second, measurement of microbial diversity would ideally complement the metabolomic data. Few studies have done so to date, and this approach represents a useful future direction (Zaura et al. 2017).

With respect to in vivo intraoral catabolism of sucrose, this work unveiled some interesting findings. A similar metabolomic approach to saccharide metabolism in plaque has been reported (Takahashi et al. 2010), but the different analytical techniques allow for different molecules to be analyzed. In the present work, salivary concentrations and outputs of molecules not always conventionally associated with glycolysis were observed, including alanine and acetoin. These differences likely serve to underline the central role of pyruvate in the oral metabolome. Pyruvate can be converted into both alanine and acetoin (March et al. 2002; Owen et al. 2002), as well entering the citric acid cycle or being converted to lactate. These latter metabolic events appeared to be associated with host sensitivity to sucrose. Lactate/pyruvate ratio in plasma is used as a medical parameter indicative of adverse metabolic events when raised. Lactate/pyruvate ratio has previously been analyzed in PS following ingestion of sugars (Kelsay et al. 1972), although the aim was to investigate how it correlated with plasma lactate/pyruvate ratio. We found a significantly higher lactate/pyruvate ratio in relatively low-sensitivity sucrose perceivers compared to high-sensitivity perceivers. Conversely, citrate/pyruvate ratios showed the opposite relationship. These metabolic differences might be explained by differences in oral microflora. Streptococci such as *Streptococcus mutans*, which are efficient oral lactate producers, feature altered or absent citric acid cycles with a limited role in energy production (Ajdić et al. 2002). Therefore, high-sensitivity sucrose perceivers could have a less lactogenic oral microbiome. Whether sensitivity to sucrose is associated with intake is controversial, involving genetic and environmental factors (Keskitalo et al. 2007; Eny et al. 2010). While some studies report no association (Cicerale et al. 2012), certain patterns of sugar consumption in the form of soft drinks have been demonstrated to reduce sucrose sensitivity (Sartor et al. 2011). Thus, as speculated (Alcock et al. 2014), taste sensitivity may be associated with the oral microbiome, leading to enhanced consumption of refined sugars, which could ultimately lead to negative oral and systemic health consequences. Future work into the nature of intraoral metabolite mediated host-microbiome interactions could potentially be adapted into functional measures of caries risk assessment. Such knowledge may also help clinicians appreciate the complex biological factors in explaining health behaviors as the dental profession collectively moves away from “patient-blaming” models of disease etiology.

## Author Contributions

A. Gardner, contributed to conception, design, data acquisition, analysis, and interpretation, drafted and critically revised the manuscript; P.W. So, G.H. Carpenter, contributed to conception, design, data analysis, and interpretation, critically revised the manuscript. All authors gave final approval and agree to be accountable for all aspects of the work.

## Supplemental Material

DS_10.1177_0022034520917142 – Supplemental material for Intraoral Microbial Metabolism and Association with Host Taste Perception Click here for additional data file. Supplemental material, DS_10.1177_0022034520917142 for Intraoral Microbial Metabolism and Association with Host Taste Perception by W. Shi, A. Gardner, P.W. So and G.H. Carpenter in Journal of Dental Research
