# Supplementary material for: Intraoral Microbial Metabolism and Association with Host Taste Perception
Source: J Dent Res. 2020 May 20;99(6):739–45. doi: 10.1177/0022034520917142 (PMC7590808; doi:10.1177/0022034520917142)
Supplement: DS_10.1177_0022034520917142 – Supplemental material for Intraoral Microbial Metabolism and Association with Host Taste Perception [file DS_10.1177_0022034520917142.pdf]

Intraoral microbial metabolism and association with host taste perception

Supplementary Material

Alexander Gardner[1,3], Po-Wah So[2], Guy H. Carpenter*[1]

1. Salivary Research, Centre for Host-Microbiome Interactions, Faculty of Dental, Oral & Craniofacial Sciences, King's College London, London, UK

2. Department of Neuroimaging, Institute of Psychiatry, Psychology and Neuroscience, King's College London, Maurice Wohl Clinical Neuroscience Institute, London, UK

3. Department of Restorative Dentistry, Dental Hospital and School, University of Dundee, Dundee, UK.

GC and PWS should be considered joint senior authors

*Corresponding author: Guy Carpenter, guy.carpenter@kcl.ac.uk, +44 20 7188 7460.

Experimental design

Experimental design in summarised in Supplementary Figure 1, below.

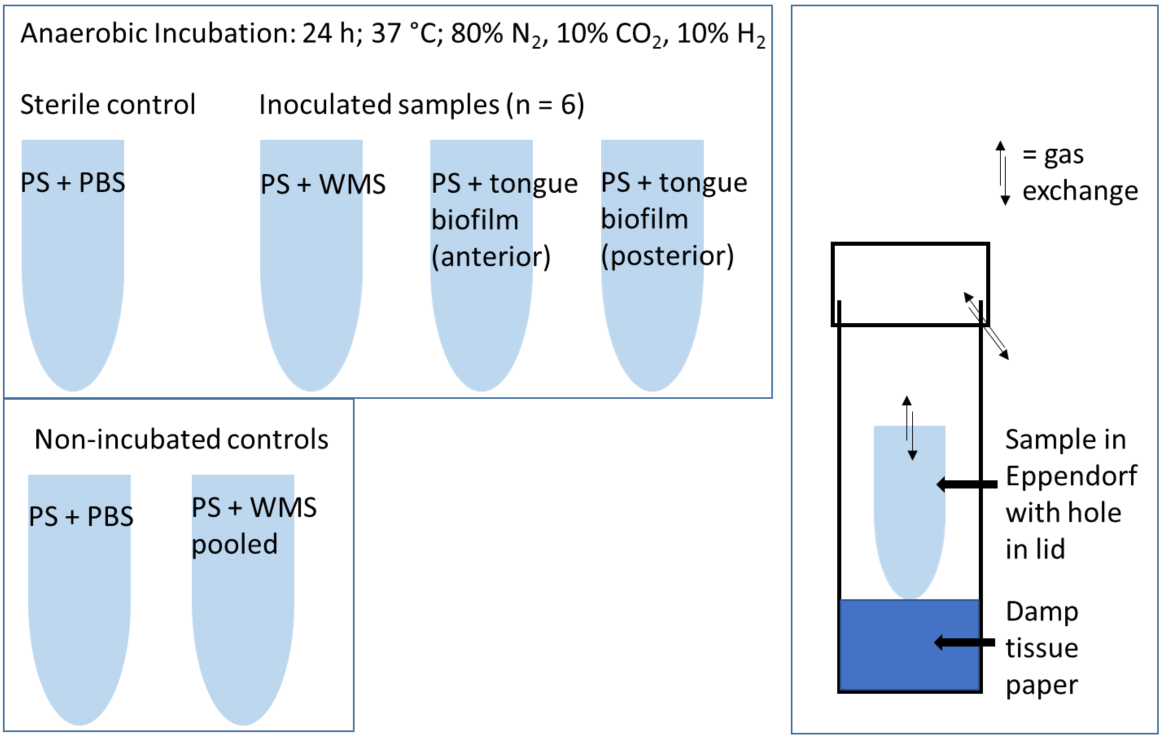

Supplementary Figure 1: An illustration of the experimental design including the different control samples and their respective incubation. On the right is the experimental setup with the sample in an Eppendorf tube with a hole in the lid for gaseous exchange inside a universal tube with the lid loosely placed to allow gas exchange with the anaerobic environment. Damp tissue paper was placed at the bottom of the tube to minimise evaporation of the sample.

Supplementary methodology

Sample analyses

Bacterial load

Final bacterial load was assessed post-incubation. Samples were vortexed to homogenise the bacterial content and sample was serially diluted ten-fold to $1:10^5$. Samples diluted to $1:10^3$ and $1:10^5$ were plated (20 µl) onto fastidious anaerobe agar with 5% defibrinated horse blood. Plates were incubated under anaerobic conditions for 48 hours, colonies counted and CFU/ml calculated.

Protein quantification

Bacterial cells were removed by centrifugation at 15,000 *g* for ten minutes at 4 °C. Samples were analysed by SDS-PAGE as previously described (Gardner and Carpenter 2019). Briefly, 12 µl buffered sample was added per lane, electrophoresed and stained with Coomassie Brilliant Blue R250 (Sigma, Gillingham, UK). Samples of the unincubated parotid saliva and incubated, PBS-inoculated parotid saliva were run on every gel. Destained gels were imaged with a ChemiDoc MP system (Biorad, Watford, UK) and analysed in ImageLab 5.2.1 (Bio-Rad Laboratories, Hercules, CA, USA). Total lane density of sample lanes and PBS-inoculated lanes relative to the unincubated parotid saliva lanes were measured. Bacterial pellets were retained for analysis confirming the protein changes observed in saliva (Supplementary Figure 2).

^{1}H-NMR spectroscopy

Centrifuged samples were prepared and analysed using internal standard as described (Gardner et al. 2018). All reagents and consumables were purchased from sigma. NMR buffer was prepared with 0.5 mM trimethylsilyl-[2,2,3,3,-$_2H^4$]-propionate (TSP) standard, 0.2 M Na_2HPO_4 and 44 mM NaH_2PO_4 in 50% deuterium oxide (D_2O) by volume. Sample (440 µl), centrifuged as for the protein analysis, was mixed with NMR buffer (110 µl) in 5 mm external diameter NMR tubes to give a final concentration of 0.1 mM TSP and 10% by volume D_2O. Using a 600 MHz spectrometer (Bruker, Karlsruhe, Germany), operating at a proton frequency of 600.2 MHz, spectra were acquired at 25 °C using a Carr-Purcell-Meiboom-Gill (CPMG) spin-echo pulse sequence with presaturation to supress macromolecule resonances from the spectra. The total echo time was 64 ms with relaxation delay of 4 s and acquisition time of 2.32 s. Following four dummy scans, 128 transients were collected with 64,000 data points and spectral width of 20 ppm (−5 to 15 ppm). Spectra were automatically phased and baseline corrected with further manual adjustment where required. The control samples described for the protein analysis were also analysed by ^{1}H-NMR spectroscopy. An additional non-incubated sample of parotid saliva with 4% pooled WMS added was prepared to control for baseline metabolite content of the inoculum.

Spectra were analysed by targeted manual quantification of known metabolites. Peak assignments were made using HMDB (hmdb.ca), Chenomx 8.3 (Edmonton, Canada) and literature values. Spectra were integrated into 0.01 ppm buckets from δ 0.7 to 8.5 ppm, excluding δ 4.5 to 5.5 ppm buckets, using MestreC (Santiago de Compostela, Spain), normalised to the standard peak, centred and Pareto scaled and then analysed by principal component analysis and k-means cluster analysis in Knime v.3.4.2 (Konstanz, Germany).

Analysis of bacterial pellets

Microbial pellets were analysed for protein content to ensure protein absent from saliva had not simply been aggregated by bacteria, something that certain oral bacterial species are capable of.

Bacterial pellets were resuspended in 500 µl sterile PBS. A protease inhibitor cocktail (Class I, Sigma) was added as instructed (1% by volume) to prevent any further potential protein degradation during preparation/analysis. Resuspended bacteria were ultrasonically lysed (ten two second pulses, with five seconds in between to prevent heat build-up) and samples of the suspension with and without pellet fragments were prepared for SDS-PAGE as described. An example gel is shown below in Supplementary Figure 2, confirming that there is negligible residual protein in the bacterial pellets and that no protein was lost during pellet preparation. Protein losses from inoculated PS were therefore due to protein catabolism rather than aggregation by bacteria.

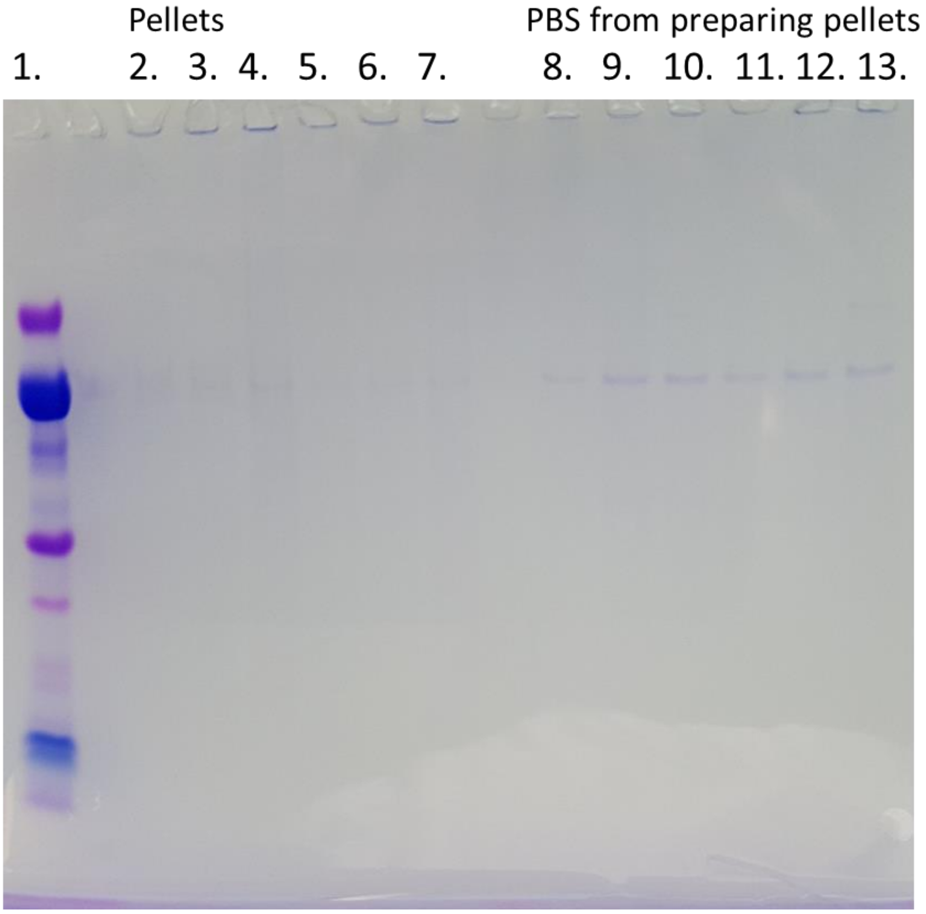

Supplementary Figure 2: Coomassie stained polyacrylamide gel showing parotid saliva (lane 1) and the lack of residual proteins within bacterial pellets (lanes 2 – 7) or PBS when preparing pellets (lanes 8 – 13). Very faint bands can be seen around the amylase bands in the PBS samples, however this does not account for the degree of protein loss observed in incubated inoculated samples. This gel appearance was typical for all samples.

Sensory Scale

An example of the generalised labelled visual analogue scale (glVAS) is shown in Supplementary Figure 3. Note the sucrose concentration is left blank at the point of rating the scale to reduce rating bias.

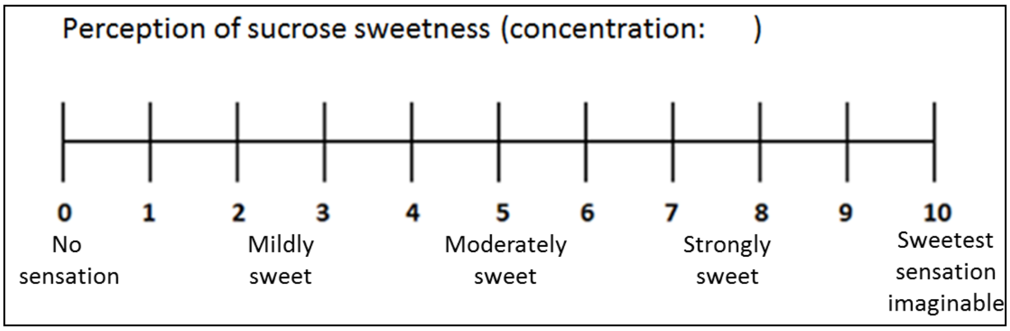

Supplementary Figure 3: Example glVAS used for assessing sucrose intensity.

Example ^{1}H-NMR spectra of PBS and bacteria inoculated parotid saliva

A comparison of the metabolic content of parotid saliva pre- and post-inoculation and incubation with oral bacteria is presented in Supplementary Figure 4. The consumption of host derived urea, citrate and lactate is visible as is the generation of SCFAs, amino acids and phenolic compounds.

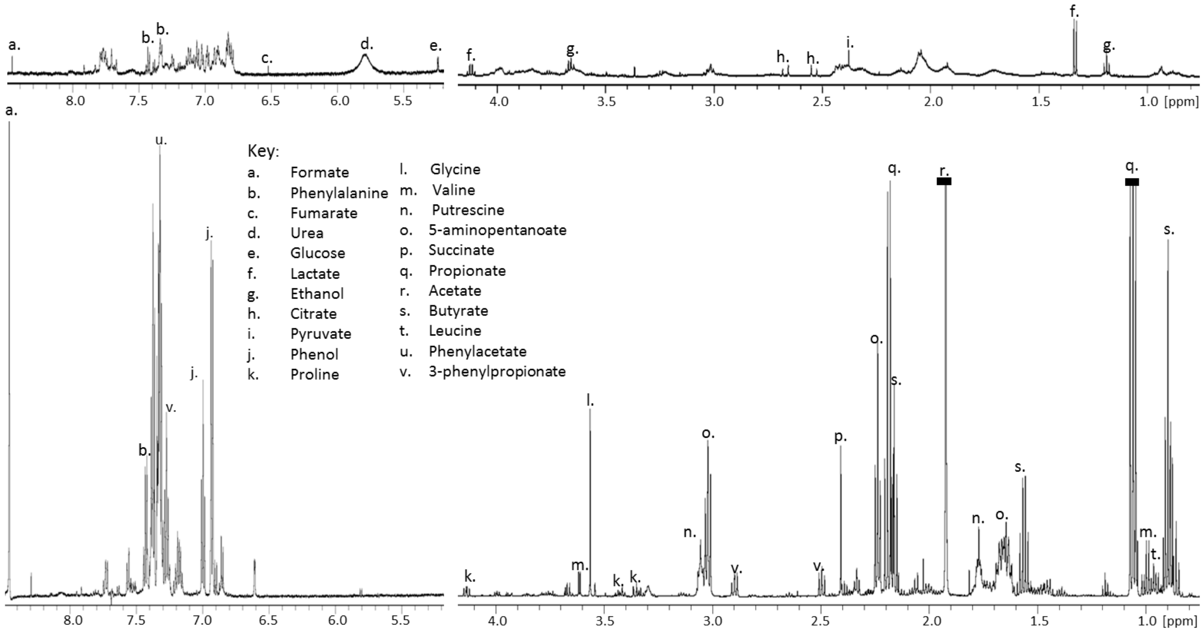

Supplementary Figure 4: Partial 1D 600 MHz CPMG ^{1}H-NMR spectra comparing PBS-inoculated parotid saliva (top spectrum) and tongue biofilm-inoculated parotid saliva (bottom spectra). The water peak region 4.2 – 5.2 ppm has been excluded. Aromatic spectral regions (5.2 – 8.5 ppm) are vertically scaled 16 times greater than aliphatic regions (0.78 – 4.2 ppm). Acetate and propionate peaks have been cropped (black bars). Spectra are to the same vertical scale.

Association between salivary protein consumption and metabolite generation by oral bacteria

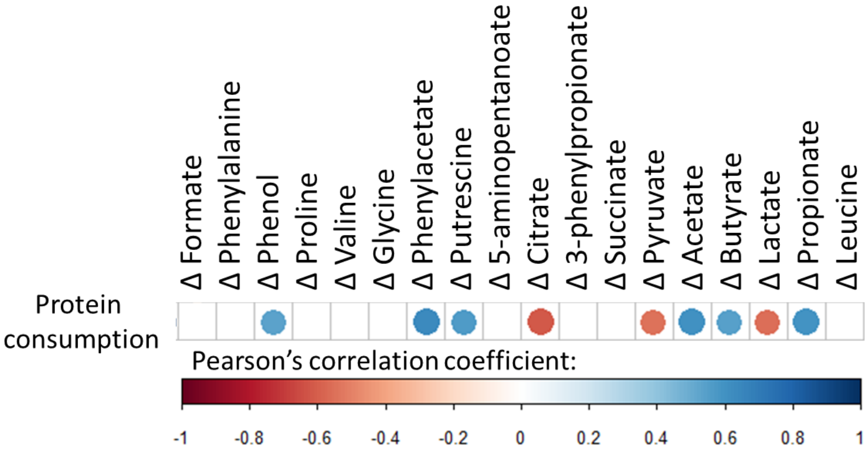

Supplementary Figure 5: A summary of the significant ($p < 0.05$) correlations between protein consumption (control lane density minus sample lane density) and change in metabolite concentration. Negative correlation indicates consumption of metabolites whereas positive correlation indicates production of metabolites.

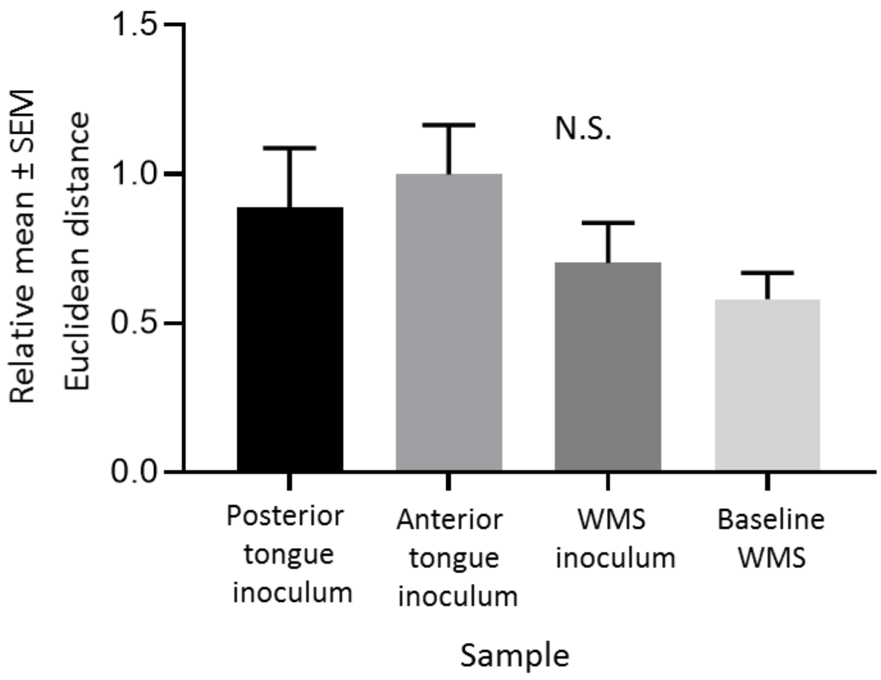

Supplementary Figure 6: Assessment of inter-individual variation of metabolite profiles of inoculated parotid saliva and participants baseline WMS. Following PCA analysis of the samples, variation was assessed by measuring Euclidean distance (weighted for PCA score of the first three axes) between participants for each sample type. A total of fifteen measurements per sample type were made (i.e. all possible pairings from six participants). Mean Euclidean distance was normalised to the anterior tongue samples, which yielded the highest inter-individual variation. ANOVA revealed no significant differences between the inter-individual variation of metabolite profiles for the different sample types.

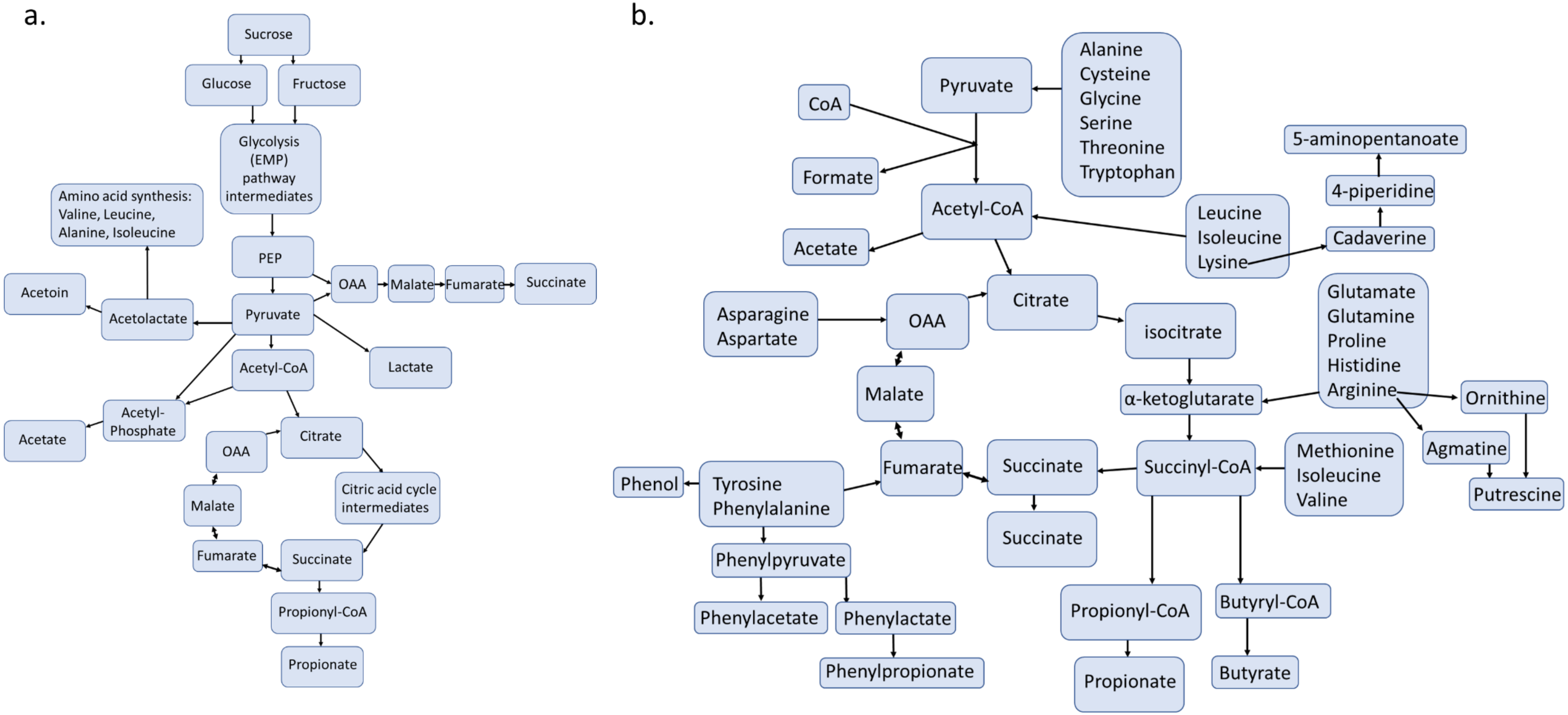

Supplementary Figure 7: A summary of oral microbial metabolic pathways. a. depicts the interactions following oral exposure to sucrose whereas b. depicts interactions in a "fasted" state where the main substrates are amino acids derived from salivary proteins. PEP = phosphoenolpyruvate, OAA = oxaloacetate, CoA = coenzyme A. Information is summarised from Takahashi (2015), Owen *et al.*, (2002), Wunderlichova *et al.,* (2014), Smith & Macfarlane (1997), Ajdic *et al.,* (2002) and Fernandez-Valero & Vendrell (2019).

Supplementary Table 1: Summary of the concentrations of metabolites consumed and generated following 24 h anaerobic incubation of parotid saliva inoculated with oral bacteria relative to inoculation with sterile PBS. Significant results ($p < 0.05$) are presented in bold. NA = statistical test could not be conducted due to total consumption of metabolite from all samples yielding a S.D. of zero; n.s. = not significant. Sample means were compared to PBS metabolite concentrations by a one-sample t-test ($n = 6$).

**Metabolite**	**Post-incubation metabolite conc. (mM) for PBS inoculated PS (n=1)**	**Mean (*S.D.*) post-incubation metabolite conc. (mM) for PS inoculated with:**			**p-value between bacterial and PBS inoculum (one-sample t-test).**		
		**Tongue biofilm (ant.) (n=6)**	**Tongue biofilm (post.) (n=6)**	**WMS (n=6)**	**Posterior tongue v. PBS control**	**Anterior tongue v. PBS control**	**WMS v. PBS control**
**Metabolites consumed**							
Urea	0.07	0.00 (*0.00*)	0.00 (*0.00*)	0.02 (*0.01*)	**NA**	**NA**	**0.01**
Lactate	0.18	0.03 (*0.02*)	0.02 (*0.01*)	0.15 (*0.01*)	**$< 10^{-5}$**	**$< 10^{-5}$**	n.s.
Citrate	0.08	0.01 (*0.01*)	0.00 (*0.00*)	0.03 (*0.02*)	**$< 10^{-5}$**	**NA**	**0.004**
Pyruvate	0.05	0.02 (*0.01*)	0.01 (*0.01*)	0.03 (*0.02*)	**$< 10^{-5}$**	**$< 10^{-5}$**	n.s.
Glucose	0.12	0.00 (*0.00*)	0.00 (*0.00*)	0.00 (*0.00*)	**NA**	**NA**	**NA**
**Metabolites generated**							
Formate	0.004	0.77 (*0.18*)	0.92 (*0.17*)	0.72 (*0.26*)	**$< 10^{-4}$**	**$< 10^{-5}$**	**0.001**
Phenylalanine	0.02	0.08 (*0.04*)	0.11 (*0.09*)	0.05 (*0.02*)	**0.015**	n.s.	**0.02**
Phenol	0.00	0.16 (*0.05*)	0.13 (*0.04*)	0.05 (*0.04*)	**$< 10^{-4}$**	**$< 10^{-4}$**	**0.03**
Proline	0.00	0.37 (*0.11*)	0.45 (*0.17*)	0.32 (*0.17*)	**$< 10^{-4}$**	**0.001**	**0.006**
Valine	0.00	0.19 (*0.10*)	0.20 (*0.12*)	0.10 (*0.06*)	**0.005**	**0.01**	**0.01**
Phenylacetate	0.00	0.09 (*0.05*)	0.05 (*0.04*)	0.01 (*0.01*)	**0.01**	**0.02**	**0.03**
Glycine	0.02	0.50 (*0.21*)	0.58 (*0.32*)	0.41 (*0.23*)	**0.003**	**0.008**	**0.008**
5-aminopentanoate	0.00	1.06 (*0.41*)	1.04 (*0.43*)	0.50 (*0.31*)	**0.001**	**0.002**	**0.01**
3-phenylpropionate	0.00	0.13 (*0.11*)	0.10 (*0.10*)	0.01 (*0.02*)	**0.03**	n.s.	n.s.
Putrescine	0.00	0.32 (0.09)	0.32 (0.06)	0.17 (0.08)	**$< 10^{-4}$**	**$< 10^{-5}$**	**0.004**
Succinate	0.04	0.05 (*0.05*)	0.09 (*0.07*)	0.09 (*0.03*)	n.s.	n.s.	**$< 10^{-4}$**
Acetate	0.01	4.70 (*1.02*)	4.19 (*1.02*)	1.85 (*0.84*)	**$< 10^{-5}$**	**$< 10^{-4}$**	**0.003**
Butyrate	0.00	0.62 (*0.25*)	0.47 (*0.25*)	0.06 (*0.01*)	**0.002**	**0.007**	n.s.
Propionate	0.00	2.33 (*0.83*)	1.97 (*0.79*)	0.37 (*0.40*)	**0.001**	**0.002**	n.s.
Leucine	0.02	0.10 (*0.06*)	0.12 (*0.09*)	0.07 (*0.04*)	**0.02**	**0.05**	**0.02**

Table 2: A summary of the salivary concentrations and output changes of salivary metabolites post-sucrose exposure, relative to water control. * - glucose is measured as the sum of α- and β-glucose quantified in the sample. Data were analysed by paired t-test (n = 18). Significant p-values ($p < 0.05$) are included in bold.

**Metabolite**	**Salivary metabolite concentration (mM):**					**Salivary metabolite output (µmol/min):**				
	**Post-control (water)**		**Post-sucrose (0.25 M)**		**p-value (paired t-test)**	**Post-control (water)**		**Post-sucrose (0.25 M)**		**p-value (paired t-test)**
	**Mean**	**S.D.**	**Mean**	**S.D.**		**Mean**	**S.D.**	**Mean**	**S.D.**	
Formate	0.068	0.118	0.056	0.063	0.54	0.128	0.193	0.122	0.138	0.89
Sucrose	0.007	0.013	18.39	9.90	**4.43×10^{-7}**	0.012	0.025	38.35	25.87	**8.16×10^{-6}**
Citrate	0.046	0.029	0.042	0.028	0.62	0.089	0.061	0.099	0.075	0.42
Succinate	0.18	0.13	0.72	0.42	**1.84×10^{-5}**	0.35	0.25	1.51	0.85	**8.54×10^{-6}**
Pyruvate	0.18	0.07	0.40	0.19	**4.31×10^{-5}**	0.33	0.15	0.84	0.38	**6.63×10^{-6}**
Acetate	4.94	2.34	4.16	1.79	**0.023**	9.53	5.09	8.87	4.09	0.38
Alanine	0.13	0.06	0.15	0.07	**0.021**	0.25	0.13	0.33	0.17	**0.003**
Acetoin	0.06	0.03	0.11	0.08	**0.004**	0.12	0.06	0.23	0.16	**0.001**
Lactate	0.53	0.53	4.42	2.84	**1.31×10^{-5}**	0.92	0.81	9.12	5.59	**1.14×10^{-5}**
Propionate	0.98	0.64	1.11	0.62	0.19	1.85	1.20	2.33	1.36	**0.047**
Butyrate	0.31	0.13	0.27	0.12	**0.005**	0.60	0.30	0.57	0.26	0.56
Glucose*	0.15	0.01	3.91	2.06	**0.006**	0.30	0.03	7.52	4.02	**0.007**

Supplementary Table 3: Summary of the salivary metabolite changes following exposure to a sucrose challenge, subdivided by participant sensitivity to the sucrose stimulus. Data are presented as concentration and output.

Metabolite concentration (mM):

**Metabolite**	**Relatively insensitive perceivers (n=9)**					**Sensitive perceivers (n=9)**				
	**Post-control (water)**		**Post-sucrose (0.25 M)**		**p-value (paired t-test)**	**Post-control (water)**		**Post-sucrose (0.25 M)**		**p-value (paired t-test)**
	**Mean**	**S.D.**	**Mean**	**S.D.**		**Mean**	**S.D.**	**Mean**	**S.D.**	
Formate	0.059	0.043	0.045	0.051	0.33	0.078	0.166	0.066	0.074	0.78
Sucrose	0.008	0.015	20.15	12.12	**0.001**	0.006	0.012	16.64	7.37	**0.0001**
Citrate	0.045	0.032	0.029	0.021	**0.025**	0.046	0.028	0.055	0.029	0.44
Succinate	0.19	0.09	0.75	0.47	**0.009**	0.18	0.17	0.70	0.39	**0.0007**
Pyruvate	0.19	0.04	0.40	0.20	**0.019**	0.17	0.10	0.40	0.20	**0.0006**
Acetate	4.77	0.76	4.00	1.35	0.15	5.11	3.31	4.32	2.22	0.10
Alanine	0.13	0.03	0.14	0.06	0.39	0.13	0.08	0.16	0.09	**0.001**
Acetoin	0.07	0.02	0.12	0.07	**0.042**	0.06	0.04	0.10	0.10	0.058
Lactate	0.55	0.39	4.63	2.59	**0.003**	0.50	0.66	4.21	3.21	**0.004**
Propionate	0.96	0.31	1.08	0.48	0.48	0.99	0.89	1.13	0.77	0.22
Butyrate	0.30	0.05	0.23	0.05	**0.015**	0.32	0.18	0.30	0.15	0.17
Glucose*	0.15	0.05	4.30	5.38	**0.05**	0.16	0.05	3.52	5.18	0.09

Salivary metabolite output (µmol/ml):

**Metabolite**	**Relatively insensitive perceivers (n=9)**					**Sensitive perceivers (n=9)**				
	**Post-control (water)**		**Post-sucrose (0.25 M)**		**p-value (paired t-test)**	**Post-control (water)**		**Post-sucrose (0.25 M)**		**p-value (paired t-test)**
	**Mean**	**S.D.**	**Mean**	**S.D.**		**Mean**	**S.D.**	**Mean**	**S.D.**	
Formate	0.116	0.102	0.103	0.126	0.63	0.141	0.262	0.142	0.154	0.99
Sucrose	0.015	0.031	31.00	14.56	**0.0002**	0.010	0.018	45.69	32.99	**0.003**
Citrate	0.075	0.049	0.057	0.047	0.11	0.104	0.070	0.142	0.076	0.07
Succinate	0.33	0.20	1.37	1.06	**0.014**	0.38	0.29	1.66	0.59	**0.0001**
Pyruvate	0.31	0.13	0.72	0.44	**0.015**	0.36	0.17	0.96	0.27	**0.0001**
Acetate	7.96	3.76	7.35	4.24	0.44	11.11	5.94	10.38	3.51	0.60
Alanine	0.22	0.12	0.28	0.21	0.15	0.27	0.14	0.38	0.12	**0.007**
Acetoin	0.11	0.06	0.24	0.20	0.06	0.12	0.07	0.23	0.11	**0.0009**
Lactate	0.86	0.61	8.82	7.25	**0.013**	0.97	1.01	9.42	3.67	**0.0001**
Propionate	1.61	0.79	2.09	1.51	0.18	2.09	1.52	2.58	1.23	0.18
Butyrate	0.48	0.17	0.43	0.20	0.20	0.71	0.36	0.72	0.24	0.90
Glucose*	0.05	0.02	3.32	5.22	0.10	0.07	0.04	2.47	2.57	0.**023**
